# Description and molecular phylogeny of a new species of *Phoronis* (Phoronida) from Japan, with a redescription of topotypes of *P. ijimai* Oka, 1897

**DOI:** 10.3897/zookeys.398.5176

**Published:** 2014-04-04

**Authors:** Masato Hirose, Ryuma Fukiage, Toru Katoh, Hiroshi Kajihara

**Affiliations:** 1 Coastal Ecosystem Restoration, International Coastal Research Center, Atmosphere and Ocean Research Institute, The University of Tokyo, Kashiwa 277-8564, Chiba, Japan; 2 Laboratory of Dead Body Science, Graduate School of Science, The University of Tokyo, Bunkyo-ku 113-0033, Tokyo, Japan; 3 Department of Natural History Sciences, Faculty of Science, Hokkaido University, Sapporo 060-0810, Hokkaido, Japan

**Keywords:** Lophophorata, 3D reconstruction, cladistic analyses, Japan, Misaki, Kyushu

## Abstract

We describe *Phoronis emigi* **sp. n.** as the eighth member of the genus based on specimens collected from a sandy bottom at 33.2 m depth in Tomioka Bay, Amakusa, Japan. The new species is morphologically similar to *P. psammophila* Cori, 1889, but can be distinguished from the latter by the number of longitudinal muscle bundles in the body wall (56–72 vs. 25–50 in *P. psammophila*) and the position of the nephridiopores (situated level with the anus vs. lower than the anus in *P. psammophila*). Using sequences of the nuclear 18S and 28S rRNA genes and the mitochondrial cytochrome *c* oxidase subunit I (COI) gene, we inferred the relationship of *P. emigi* to other phoronids by the maximum likelihood method and Bayesian analysis. The analyses showed that *P. emigi* is closely related to *P. hippocrepia* Wright, 1856 and *P. psammophila* Cori, 1889. We describe the morphology of the topotypes and additional material for *P. ijimai* Oka, 1897. Neither our morphological observations of *P. ijimai*, nor the phylogenetic analyses based on 18S and COI sequences, contradicts that *P. vancouverensis* Pixell, 1912 is conspecific with *P. ijimai*, a synonymy that has long been disputed.

## Introduction

Phoronids, or horseshoe worms, are exclusively marine, sedentary, vermiform animals with a crown of ciliated tentacles, the lophophore, used in suspension feeding. They comprise the small phylum Phoronida, which currently contains two genera, *Phoronis* Wright, 1856 and *Phoronopsis* Gilchrist, 1907, with seven and three species, respectively (Emig 2007). Phoronid species are morphologically well defined, primarily on the basis of the arrangement and pattern of the body-wall musculature, nephridia, and lophophore in adults (e.g., Emig 1974, 1979, 1982). They produce characteristic actinotroch larvae, and most species have a cosmopolitan distribution (Emig 1982, Zimmer 1991).

For over the last half century, no new species of phoronids have been established, although the current species diversity is likely to have been underestimated (Santagata and Zimmer 2002), with *Phoronis pallida* Silén, 1952 and *Phoronopsis californica* Hilton, 1930 being the most recently described valid species in each genus (Silén 1952, Hilton 1930). More recently described nominal species have been regarded as invalid, junior synonyms of older names based on morphological concordance: *Phoronis svetlanae* Temereva & Malakov, 1999 as synonymous with *Phoronis ijimai* Oka, 1897 (Emig 2007), and *Phoronopsis malakhovi* Temereva, 2000 with *Phoronopsis harmeri* Pixell, 1912 (Emig 2003). Since DNA sequence data have been obtained for almost all valid species in the phylum (e.g., Santagata and Cohen 2009, and references therein), sequences from *Phoronis svetlanae* and *Phoronopsis malakhovi* would have helped either to discriminate these species from congeners or to corroborate the proposed synonymies.

One of the unsettled taxonomic issues in phoronid systematics is whether or not *Phoronis ijimai* Oka, 1897 (type locality: Misaki, Japan) is conspecific with *Phoronis vancouverensis* Pixell, 1912 (type locality: Vancouver, Canada). Emig (1971a, b, 1974, 1977, 1982, 2007) synonymized these two nominal species based on similarity in various anatomical features in adults. Santagata and Zimmer (2002), however, avoided drawing a definitive conclusion on this synonymy, arguing that the late and competent larval stages described by Zimmer (1964) for *Phoronis vancouverensis* were not recorded for *Phoronis ijimai* in developmental observations by Ikeda (1901) and Wu and Sun (1980). Most of the DNA sequences from species in this complex currently deposited in GenBank are registered under the name *Phoronis vancouverensis*, and all are derived from specimens collected in the northeastern Pacific, at localities closer to Vancouver than to Misaki: Friday Harbor, WA (Fuchs et al. 2009, Sperling et al. 2011); Monterey, CA (Cohen 2000, Mallatt and Winchell 2002); and Los Angeles, CA (Erber et al. 1998). For some sequences, the locality of origin is not reported in GenBank (Halanych et al. 1995, Passamaneck and Halanych 2006, Bourlat et al. 2008). On the other hand, no sequence data have been reported for *Phoronis ijimai*, either from its type locality or a reasonably close locality in the northwestern Pacific. Undoubtedly, this has in part contributed to the continuing dispute over synonymy.

In this paper, we 1) describe a new phoronid species from Japan, which differs from all the previously known species in adult morphology; 2) reconstruct the phylogeny of representative phoronids, including the new species, based on DNA sequences of the nuclear 18S and 28S rRNA genes (hereafter, 18S and 28S, respectively), and the mitochondrial cytochrome *c* oxidase subunit I gene (COI); 3) describe topotypes of *Phoronis ijimai* from Misaki, Sagami Bay, and discuss the synonymy with *Phoronis vancouverensis* in the context of adult morphology and the molecular phylogeny; and 4) provide a key to the Japanese phoronid species.

## Material and methods

### Sampling

**Figure 1.**
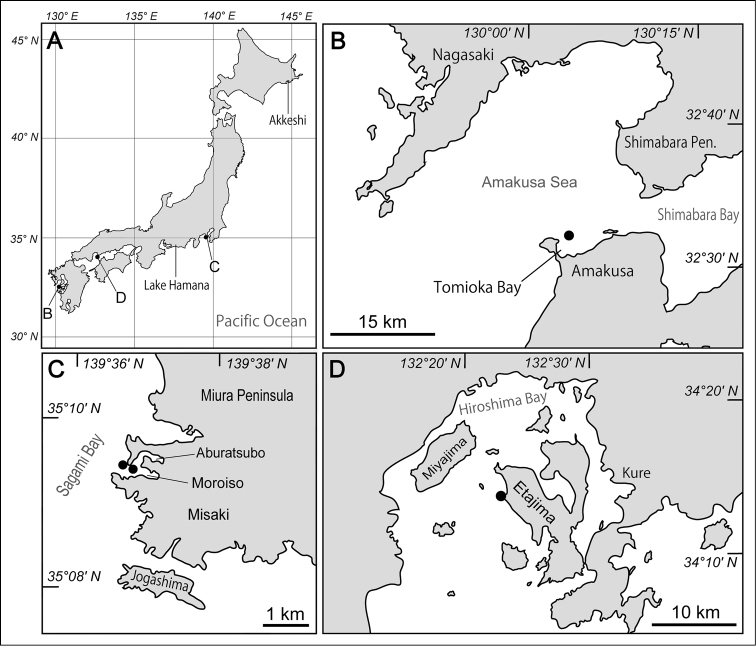
Maps showing the locations of collecting sites. **A** Map of Japan showing the collecting localities and the locations of Lake Hamana and Akkeshi **B** enlargement of west-central Kyushu, with the solid circle indicating the collecting site at Amakusa **C** enlargement of the southwestern part of the Miura Peninsula, with solid circles indicating the topotype collecting sites (type localities) of *Phoronis ijimai* Oka, 1897 at Misaki, Sagami Bay **D** enlargement of Hiroshima Bay, with the solid circle indicating an additional collecting site for *Phoronis ijimai* at Etajima.

A sediment sample was obtained with a Smith-McIntyre grab having an aperture of 25 cm × 25 cm, from a sandy bottom at 33.2 m depth (32°32'27"N, 130°03'17"E) in Tomioka Bay, Amakusa, Kumamoto, Japan (Fig. 1A, 1B) on 26 November 2009 by Keiichi Kakui, Hiroshi Yamasaki, and Shushi Abukawa on board the research and training vessel *Seriola* of the Amakusa Marine Biological Laboratory (AMBL), Kyushu University. The sediment was agitated and stirred in a bucket with seawater and the supernatant was decanted; specimens suspended in the supernatant were collected with a sieve having a 0.3-mm mesh size. Of the 560 specimens obtained, most were fixed in 10% formalin seawater, and the rest were placed directly in 99% EtOH.

Topotypes of *Phoronis ijimai* were collected in Moroiso Bay, from a pier (≈35°09'28"N, 139°36'44"E) in front of the Misaki Marine Biological Station (MMBS), The University of Tokyo, Kanagawa, Japan (Fig. 1C) on 10 May 2012 by Hisanori Koutsuka, and from a rocky shore (≈35°09'32"N, 139°36'40"E) beside Arai Beach, Sagami Bay, near MMBS on 7 May 2012 by Mayumi Masuda. Additional specimens of *Phoronis ijimai* were collected at Irukabana (≈34°13'42"N, 132°23'03"E), Etajima Island, Hiroshima, Japan (Fig. 1D) on 13 February 2011 by Daisuke Ueno.

### Morphological observation

Measurements of the lophophore and body size were taken from digital photographs with ImageJ 1.37v software (Rasband 1997–2011, Abramoff et al. 2004). For observation of internal morphology, specimens were dehydrated in an ethanol series, cleared in *n*-butanol, embedded in paraffin, sectioned at a thickness of 5–6 μm, and stained with hematoxylin-eosin (HE). DeltaViewer 2.1.1 software (Wada et al. 2005) was used to construct three-dimensional images of the nephridium. All the type and voucher specimens have been deposited in the National Museum of Nature and Science, Tsukuba, Japan (NSMT).

### DNA extraction and PCR amplification

Total genomic DNA was extracted from one of the ethanol-fixed specimens of the new species, as well as one of the topotypes of *Phoronis ijimai* (NSMT-Te 881), using a DNeasy Blood and Tissue Kit (Qiagen), following the manufacturer’s protocol. The 18S gene was amplified with three primer sets: 1F/4R, 3F/18sbi, and 18Sa2.0/9R (Giribet et al. 1996, Whiting et al. 1997). The 28S fragment was amplified with primer set LSU5/LSU3 (Littlewood 1994). The COI fragment was amplified with the primer pair LCO1490/HCO2198 (Folmer et al. 1994). PCR reactions were performed with *ExTaq* (TaKaRa). Conditions for hot-start thermal cycling were 2 min at 94 °C; 35 cycles of 45 sec at 94 °C, 45 sec at 50 °C, and 90 sec at 72 °C; and 7 min at 72 °C. PCR products were visualized on a 1% agarose gel and purified according to the method of Boom et al. (1990) with some modifications (Kobayashi and Tachi 2009, Kobayashi et al. 2009). Cycle sequencing was performed with BigDye Terminator 3.1 (Life Technologies). The PCR primers were used for sequencing reactions, together with two additional 28S primers, D2F (Littlewood 1994) and a truncated version (Thollesson and Norenburg 2003) of 28z (Hillis and Dixon 1991). Both product strands were sequenced with an ABI 3130 Genetic Analyzer (Life Technologies). Chromatograms were edited and overlapping sequence fragments were assembled by using ATGC 4.0.6 (GENETYX). The sequences have been deposited with DDBJ/EMBL/GenBank under accession numbers AB621913–AB621915 for the new species and AB752304–AB752305 for *Phoronis ijimai* (Table 1).

**Table 1.** Taxa included in the phylogenetic analyses and GenBank accession numbers for sequences. Sequences obtained in this study are in **bold.**

Species	COI	18S	28S	Reference
*Phoronis emigi* sp. n.	**AB621915**	**AB621913**	**AB621914**	this study
*Phoronis architecta*	AY368231.1	AF025946	EY334109	a (COI), b (18S), c (28S)
*Phoronis australis* (New Caledonia)	EU484457	AF202111	EU334110	c (COI, 28S), d (18S)
*Phoronis australis* (Japan)	EU484458	EU334122	EU334111	c
*Phoronis australis* (Australia)	—	EU334123	EU334112	c
*Phoronis australis* (Spain)	—	AF119079	—	e
*Phoronis hippocrepia*	EU484459	AF202112	AY839251	c (COI), d (18S), f (28S)
*Phoronis ijimai*	**AB752304**	**AB752305**	—	this study
*Phoronis muelleri*	EU484460	EU334125	EU334114	c
*Phoronis ovalis*	EU484461	EU334126	EU334115	c
*Phoronis pallida*	—	EU334127	EU334116	c
*Phoronis vancouverensis*/*ijimai*	EU484462	AF202113	AF342797	c (COI), d (18S), g (28S)
*Phoronopsis californica*	EU484463	EU334129	EU334118	c
*Phoronopsis harmeri*	EU484464	EU334130	EU334119	c
*Phoronopsis viridis*	EU484465	AF123308	EU334120	c
*Novocrania anomala*	—	AY842018	AY839245	f
*Discinisca* cf. *tenuis*	—	AY842020	AY839248	f
*Glottidia pyramidata*	—	U12647	AY839249	f (28S), h (18S)

**a** Helfenbein and Boore (2004); **b** Cohen et al. (1998); **c** Santagata and Cohen (2009); **d** Cohen (2000); **e** Giribet et al. (2000); **f** Cohen and Weydmann (2005); **g** Mallatt and Winchell (2002); **h** Halanych et al. (1995)

### Morphological analyses

From the literature (Emig 1974, Santagata and Cohen 2009) and our own data, we tabulated 32 morphological and reproductive characters (Suppl. material 1) among 11 phoronid species. Based on this data matrix, we performed three different analyses using Mesquite version 2.75 (Maddison and Maddison 2011): 1) a cluster analysis with single-linkage method based on distances between taxa calculated from the data matrix; 2) a morphology-based cladistic analysis; and 3) a most-parsimonious reconstruction of ancestral characters. For the cladistic analysis, a heuristic search was conducted with tree length criterion and rearrangement by subtree pruning and regrafting (SPR); all trees were rooted with *Phoronis ovalis* Wright, 1856 as the outgroup based on the results of Santagata and Cohen (2009). The ancestral character reconstruction was carried out based on the maximum-likelihood tree based on concatenated COI–18S–28S dataset (see below) for the 21 adult morphological characters.

### Molecular phylogeny

We checked validity of the yielded COI sequences to prevent the isolation of nuclear encoded mitochondrial psuedogenes (NUMTS) instead of true mitochondrial sequences before phylogenetic analyses. We regarded the consistently yielded fine single peaks for all the analysed sites in chromatograms and including neither indel nor stop codon as the criteria for judging the safely rejection of the possibility for the contamination of NUMTS.

The COI, 18S, and 28S sequences obtained for the new species were aligned with those from other phoronids deposited in GenBank (Table 1) using Clustal W (Thompson et al. 1994) implemented in Seaview 4.2.5 (Gouy et al. 2010) and/or MEGA 5.05 (Tamura et al. 2011). The alignment was performed gene by gene, before concatenated data sets were generated. These sequences were analyzed both independently and as concatenated data sets.

Maximum likelihood (ML) analyses was performed with MEGA 5.05. For ML, the best-fit model for all data sets determined by the AICc implemented in MEGA 5.1 was GTR+G+I (general time reversible [Tavaré 1986] with gamma-distributed rates and invariant rates among sites). Optimal ML trees were found by a nearest neighbor interchanges (NNI) search, starting with a tree topology generated by the BIONJ method (Gascuel 1997) using maximum composite likelihood (MCL) distances (Tamura et al. 2004). One-thousand bootstrap pseudoreplicates were analyzed to obtain nodal support values.

Bayesian analyses were performed by using MrBayes 3.1.2 (Ronquist and Huelsenbeck 2003). The best-fit substitution model was GTR+G+I model, determined from AICc tests in MrModeltest 2.3 (Nylander 2004) and PAUP* 4.0b10 (Swofford 2003). A Markov-Chain Monte-Carlo (MCMC) search was performed with four chains, each of which was run for 1,000,000 generations. Trees were sampled every 100 generations, and those from the first 250,000 generations were discarded as burn-in, ensuring that a stable likelihood had been reached. Trace files generated by Bayesian MCMC runs were inspected in TRACER 1.5.0 (Rambaut and Drummond 2007) to check that the number of sampling generations and effective sample sizes were large enough for reliable parameter estimates. A consensus of sampled trees was computed, and the posterior probability for each interior node was obtained to assess the robustness of the inferred relationships.

The 18S and 28S trees were rooted with three brachiopods (*Novocrania anomala*, *Discinisca* cf. *tenuis*, and *Glottidia pyramidata*) as outgroup taxa (Cohen and Weydmann 2005, Halanych et al. 1995). The COI tree was rooted with *Phoronis ovalis* Wright, 1856 as the outgroup based on the results of Santagata and Cohen (2009).

Since most of the sequences used in this study were obtained from GenBank, we used the original specific names in GenBank given by the previous authors (Halanych et al. 1995, Cohen et al. 1998, Cohen 2000, Giribet et al. 2000, Mallatt and Winchell 2002, Helfenbein and Boore 2004, Cohen and Weydmann 2005, Santagata and Cohen 2009) in Table 1. However, to make the discussion clear, we also indicate taxonomically valid specific names in our results and discussion, i.e., *Phoronis ijimai* instead of *Phoronis vancouverensis*, *Phoronis psammophila* instead of *Phoronis architecta*, and *Phoronopsis harmeri* instead of *Phoronopsis viridis*.

## Taxonomy

### Phoronis ijimai

Oka, 1897

http://species-id.net/wiki/Phoronis_ijimai

**Figure 2.**
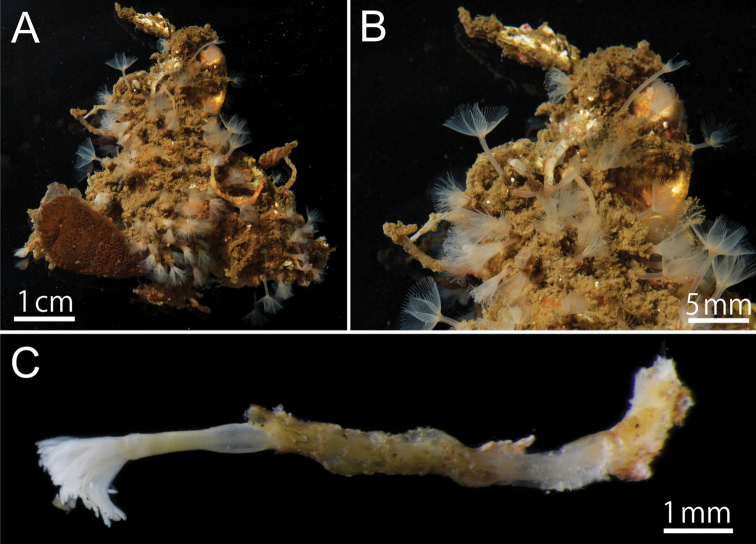
*Phoronis ijimai* Oka, 1897, NSMT-Te 879. **A** Living individuals collected from the pier of Misaki Marine Biological Station **B** enlargement of living individuals **C** preserved individual (10% formalin seawater) with a transparent cylindrical tube.

[Japanese name: Hime-houkimushi] Figures 2 [Fig F3] [Fig F4] [Fig F5] [Fig F6] –7

**Figure 3. F3:**
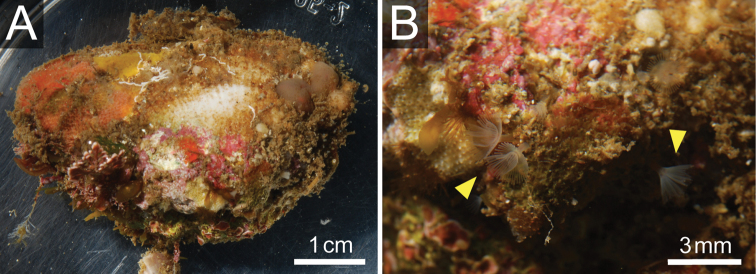
*Phoronis ijimai* Oka, 1897, NSMT-Te 880. **A** Living bivalve (*Barbatia* sp.) with various sessile organisms **B** Living *Phoronis ijimai* on the shell (arrowheads).

**Figure 4. F4:**
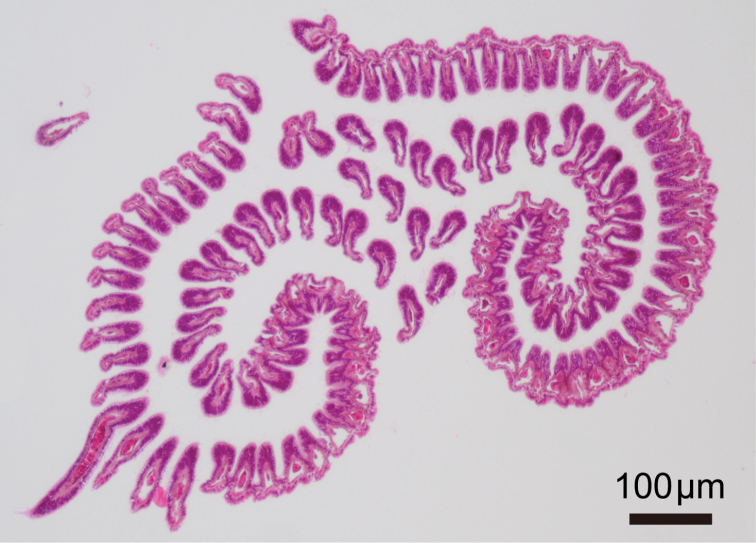
*Phoronis ijimai* Oka, 1897, NSMT-Te 885, transverse section through basal part of lophophore.

**Figure 5. F5:**
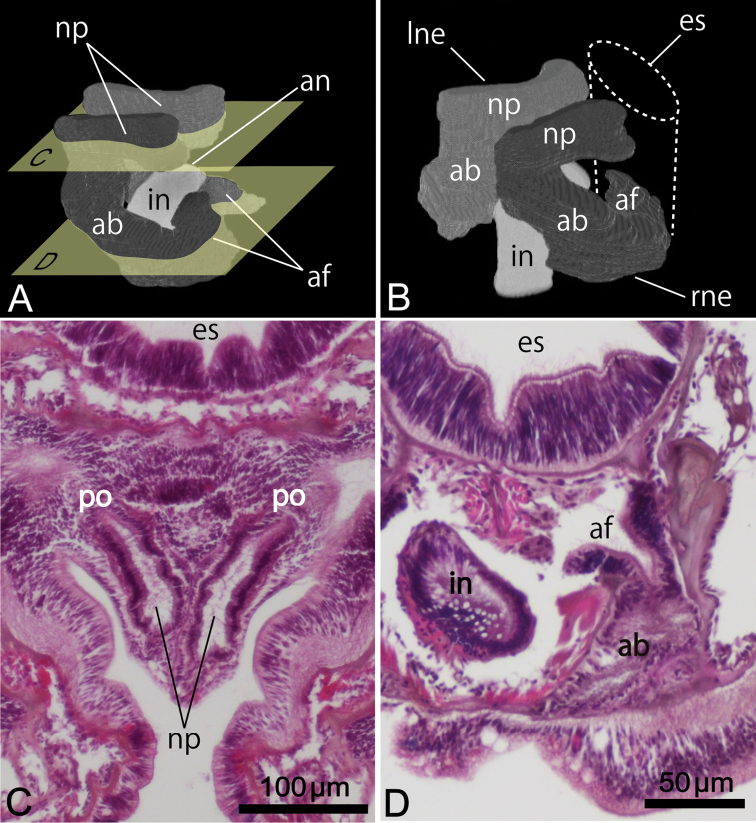
Reconstructed three-dimensional images and transverse sections of the nephridium of *Phoronis ijimai* Oka, 1897, from NSMT-Te 886 (**A, B, D**) and NSMT-Te 884 (**C**). **A** Lateral view, showing the long nephridial papillae above the anus **B** dorsolateral view, showing the offset arrangement of the nephridia, with the curved ascending branch and large anal funnel extending toward the esophagus **C** transverse section through the nephridial papilla, showing the nephridiopore **D** transverse section through the ascending branch, showing the large anal funnel opening toward the esophagus, Abbreviations: **ab** ascending branch; **af** anal funnel; **an** anus; **es** esophagus; **in** intestine; **lne** left nephridium; **np** nephridial papilla; **p** nephridiopore; **rne** right nephridium. Planes **C** and **D** in panel **A** indicate the positions of the transverse sections in **C** and **D.**

Phoronis ijimai Oka, 1897, 147–148.
Phoronis vancouverensis Pixell, 1912, 257–271, figs 1–5.
Phoronis svetlanae Temereva & Malakov, 1999, 627–630, figs 1, 3, 4.
Phoronis hippocrepia ? : Uchida and Iwata 1955, 1–3, text-figs 1, 2, pl. 1, figs A–D.

#### Material examined.

Five series of transverse sections and 34 whole specimens. NSMT-Te 878, several specimens, fixed and preserved in 10% formalin, collected at Etajima Island; NSMT-Te 879, several individuals, fixed and preserved in 10% formalin, collected in Moroiso Bay, attached to the pier in front of MMBS; NSMT-Te 880, several individuals on a living shell of *Barbatia* sp. (Mollusca: Bivalvia), collected in Sagami Bay; NSMT-Te 881, same data as NSMT-Te 879; NSMT-Te 882, same data as NSMT-Te 880; NSMT-Te 883, 6-μm transverse section stained with HE, collected at Etajima Island; NSMT-Te 884, same data as NSMT-Te 883; NSMT-Te 885, 6-μm transverse sections stained with HE, collected in Moroiso Bay; NSMT-Te 886, same data as NSMT-Te 885; NSMT-Te 887, 6-μm transverse sections stained with HE, collected in Sagami Bay.

#### Description.

Body except lophophore 2.40–16.83 mm in length (avg. 5.87±4.04 mm, n = 34; average of topotypes 9.55±4.78 mm, n = 12); 0.49–0.90 mm in diameter at ampula (avg. 0.64±0.11 mm, n = 34; average of topotypes 0.59±0.12 mm, n = 12); white and translucent in living state (Figs 2A, 2B, 3), yellowish white after fixation (Fig. 2C). Lophophore horseshoe-shaped, without significant coiling (Fig. 4); 0.87–3.11 mm in length (avg. 2.17±0.55 mm, n = 34; average of topotypes 1.66±0.49 mm, n = 12), 0.27–0.99 mm in diameter at its base (avg. 0.61±0.17 mm, n = 34; avg. of topotypes 0.43±0.09 mm, n = 12); tentacles 106–151 in number (avg. 129±18, n = 7; avg. of topotypes 110±5, n = 3). Inhabits a transparent cylindrical tube either encrusting or burrowing in hard substrates (Fig. 2C).

Nephridium 162.00–204.00 μm in height (avg. 183.00±29.70 μm, n = 2), with straight nephridial papilla and curved ascending branch (Fig. 5A, 5B). Descending branch absent. Ascending branch with single chamber. Nephridial papilla situated beside anus, 294.24–324.91 μm in length (avg. 309.57±21.69 μm, n = 2); nephridiopore situated on nephridial papilla opening above (in living orientation) anus level (Fig. 5A, 5C). Ascending branch offset along body axis near intestine, with its lower end extending toward esophagus (Fig. 5B, 5D); 277.55–323.49 μm in length (avg. 300.52±32.49 μm, n = 2). Two nephridial funnels present; anal funnel larger than oral funnel. Anal funnel large (avg. 69.00±4.24 μm in height, 45.77±3.15 μm in width at base, 111.94±16.48 μm in maximum width at tip; n = 2), its aperture located at lower end of ascending branch. Oral funnel small (avg. 20.01±1.40 μm in diameter, n = 2), its aperture opening on lateral surface of ascending branch, situated slightly lower than anal funnel.

**Figure 6. F6:**
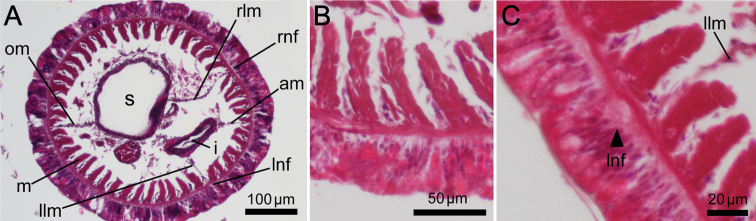
*Phoronis ijimai*. **A** NSMT-Te 886, transverse section through the posterior part of the body, showing four mesenteries and the position of the giant nerve fibers **B** NSMT-Te 885, enlargement showing longitudinal muscles of the bushy type **C** NSMT-Te 885, enlargement of the left giant nerve fiber situated at the base of the left lateral mesentery. Abbreviations: **am** anal mesentery; **i** intestine; **llm** left lateral mesentery; **lnf** left giant nerve fiber; **m** longitudinal muscle; **om** oral mesentery; **rlm** right lateral mesentery; **rnf** right giant nerve fiber; **s** stomach.

Body-wall longitudinal muscles of generally bushy type (Fig. 6A, 6B) but sometimes feathery in lower part of body; 45–50 in number, arranged in following formula (Selys-Longchamps 1907):

Composite formula


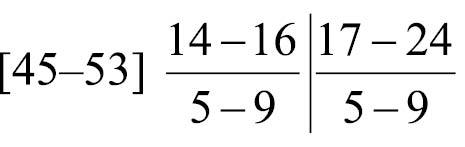


Mean formula


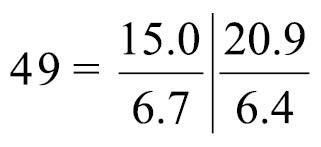


(n = 7 sections from 3 individuals)

Left and right lateral mesenteries present (Fig. 6A). Two giant nerve fibers present; left giant nerve fiber 3.16–10.61 μm in diameter (avg. 6.72±3.27 μm, based on eight sections from different parts of the body, from two individuals), situated at base of left lateral mesentery (Fig. 6C); right giant nerve fiber 2.47–7.81 μm in diameter (avg. 4.55±2.15 μm, based on nine sections of different parts of the body from two individuals), situated at base of right lateral mesentery. Esophageal valve absent.

**Figure 7.**
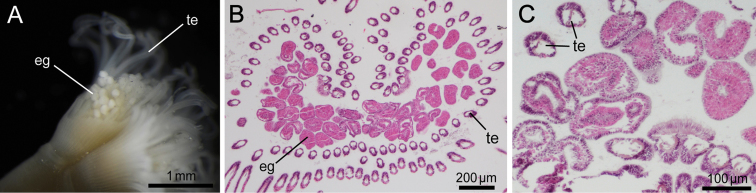
*Phoronis ijimai*. **A** NSMT-Te 878, eggs brooded in the lophophore (some tentacles have been removed) **B** NSMT-Te 884, transverse section through the basal part of the lophophore, showing mature eggs on the basal nidamental glands **C** NSMT-Te 883, enlargement of brooded eggs, showing various developmental stages. Abbreviations: **eg** egg; **te** tentacle.

Hermaphroditic; early-stage ova and spermatocytes found beside lateral blood vessel. Brooded eggs observed in specimens from Hiroshima (Fig. 7A, 7B, 7C); embryos of various developmental stages brooded on basal nidamental glands on lophophore (Fig. 7C).

#### Distribution and habitat.

*Phoronis ijimai* is widely distributed in the North Pacific, along the coasts of North America, Canada, Japan, and Russia, including the Sea of Japan (Emig 1971a, 1974, Emig and Golikov 1990, Temereva and Malakhov 1999). *Phoronis ijimai* has been reported from hard substrates such as rocks, bivalve shells, and wood, and also from a sandy bottom; it often forms dense populations, up to about 15,000 individuals per m^2^ (Emig 1974).

#### Remarks.

Our topotype material of *Phoronis ijimai* collected from Misaki perfectly agrees with previous morphological accounts of this species (Oka 1897, Emig 1971a, 1974) in the following characters: 1) the long nephridial papilla and the large anal funnel of the nephridium, 2) the small diameter of the two giant nerve fibers, 3) the number of longitudinal muscles in the right oral and both anal coeloms, and 4) the brooding of embryos on lophophoral organs. These characters also agree with the description of *Phoronis hippocrepia*, but differ in 1) the large number of longitudinal muscles in the right oral coelom, and 2) the single chamber in the ascending branch of the nephridium. Our topotypes of *Phoronis ijimai* also match the description of *Phoronis vancouverensis* (Pixell 1912, Emig 1971a, 1974). While our specimens have slightly fewer longitudinal muscles in the right anal and left oral coeloms compared to the original description of *Phoronis vancouverensis* by Pixell (1912) and the revised description of *Phoronis ijimai* by Emig (1974), respectively, the numbers are within the range of variation in *Phoronis ijimai* (Emig 1974). The topotypes had fewer tentacles, probably due to the smaller size of the body and lophophore.

### Phoronis emigi sp. n.

http://zoobank.org/51F10DA8-DE79-4537-86E7-DE2F1CBC1B56

http://species-id.net/wiki/Phoronis_emigi

**Figure 8.**
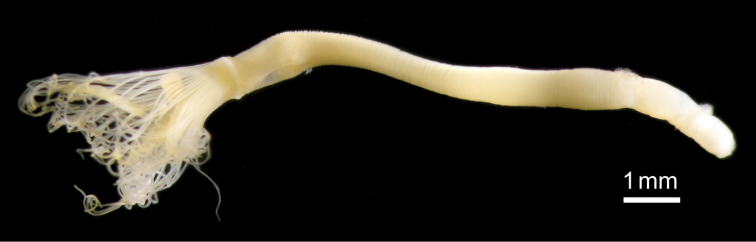
*Phoronis emigi* sp. n., NSMT-Te 714 (holotype), photographed in the preserved state (10% formalin seawater) before sectioning.

[New Japanese name: Amakusa-houkimushi] Figures 8 [Fig F9] [Fig F10] –11

#### Material examined.

Eleven series of transverse sections and two series of longitudinal sections, and nine whole specimens. *Holotype*: NSMT-Te 714, 5-μm transverse sections stained with HE. *Paratypes*: NSMT-Te 703–708, seven intact specimens, fixed and preserved in 10% formalin seawater; NSMT-Te 711–713, 715–721, 5-μm transverse sections stained with HE; and NSMT-Te 722, 723, 5-μm longitudinal sections stained with HE. *Other material examined*: NSMT-Te 709, 710, two intact specimens.

#### Etymology.

The specific name, a masculine noun in the genitive case, is in honor of the French researcher Dr. Christian C. Emig for his remarkable contributions to lophophorate systematics.

#### Description.

**Figure 9. F9:**
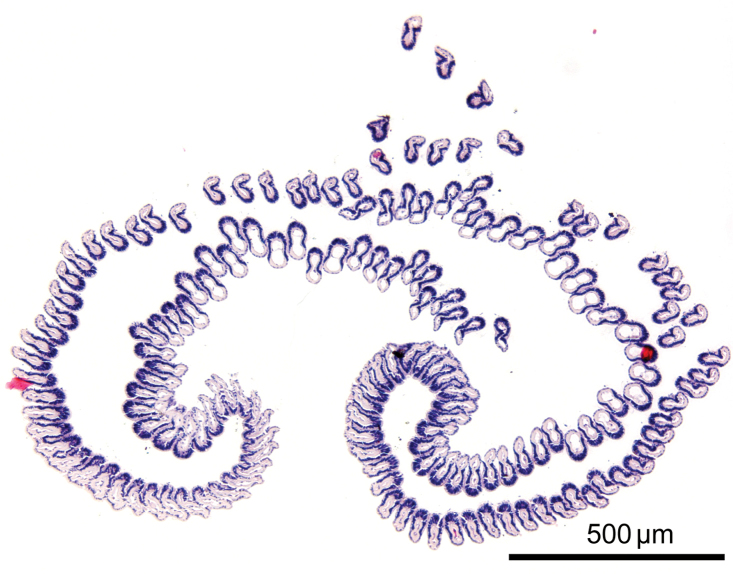
*Phoronis emigi* sp. n., NSMT-Te 713 (paratype), transverse section through the basal part of the lophophore.

Body except lophophore 4.42–20.06 mm in length (holotype 9.67 mm; avg. 10.87±4.70 mm, n = 10); 0.34–0.66 mm in diameter at ampula (holotype 0.39 mm; avg. 0.47±0.10 mm, n = 9); reddish in living state, yellowish white after fixation (Fig. 8). Lophophore horseshoe-shaped, without significant coiling (Fig. 9); 2.00–3.51 mm in length (holotype 3.18 mm; avg. 2.77±0.52 mm, n = 10), 0.54–0.76 mm in diameter at base (holotype 0.68 mm; avg. 0.67±0.07 mm, n = 10); tentacles 136–170 in number (holotype 137; avg. 147±13.17, n = 6).

**Figure 10. F10:**
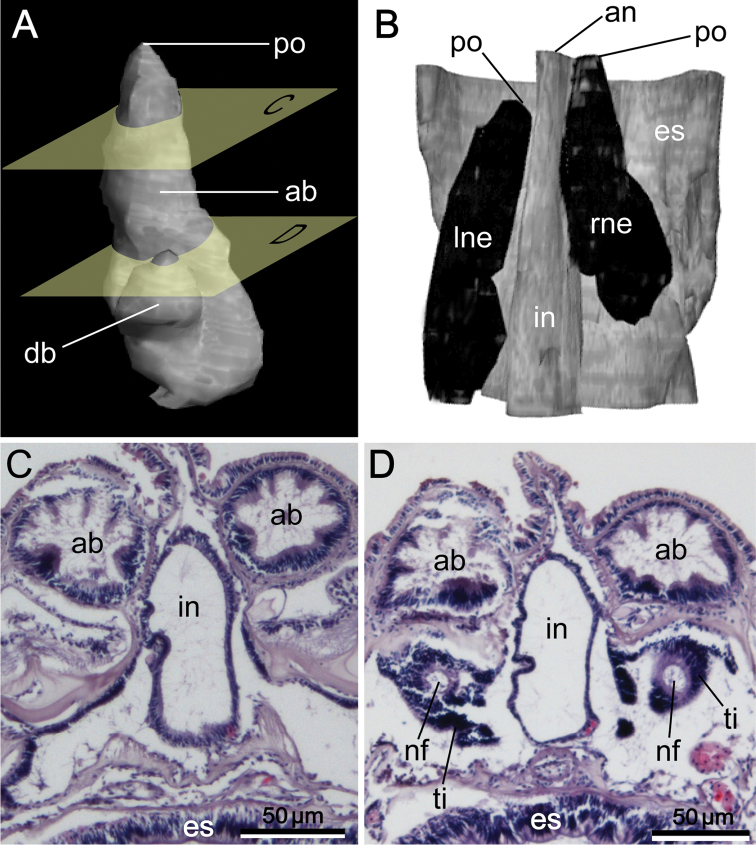
Reconstructed three-dimensional images and transverse sections of the nephridium of *Phoronis emigi* sp. n., based on NSMT-Te 721 (paratype). **A** Lateral view, showing the different lengths of the ascending and descending branches **B** dorsal view, showing the offset arrangement of the nephridia, with the nephridiopores at different levels along the body axis **C** transverse section through the ascending branch **D** transverse section through the tip of the descending branch, showing the nephridial funnels. Abbreviations: **ab** ascending branch; **an** anus; **db** descending branch; **es** esophagus; **in** intestine; **lne** left nephridium; **nf** nephridial funnel; **p** nephridiopore; **rne** right nephridium; **ti** funnel tissue. Planes **C** and **D** in panel **A** indicate the positions of the transverse sections in **C** and **D.**

Nephridium 205.00–324.00 μm in length (holotype 310 μm; avg. 276.78±38.69 μm, n = 5), with straight ascending branch (ab) and short descending branch (db) (Fig. 10A), ab/db length ratio 3.5 (n = 5). Ascending branch with single chamber (Fig. 10C). Nephridiopore situated on anal papilla. Tip of ascending branch (i.e., nephridiopore) lying against intestine. Nephridia slightly offset along body axis (Fig. 10B); left nephridiopore lower (in living orientation) than anus, right nephridiopore same level as anus. Single nephridial funnel present, with aperture at tip of descending branch (Fig. 10D).

**Figure 11.**
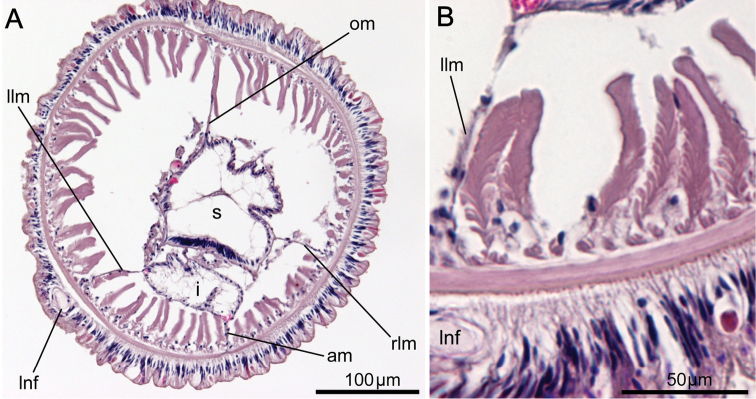
*Phoronis emigi* sp. n., NSMT-719 (paratype). **A** Transverse section through the posterior part of the body, showing four mesenteries and the position of the giant nerve fiber **B** enlargement of longitudinal muscles of the long feathery type. Abbreviations: **am** anal mesentery; **lnf** left giant nerve fiber; **i** intestine; **llm** left lateral mesentery; **om** oral mesentery; **rlm** right lateral mesentery; **s** stomach.

Body-wall longitudinal muscles of feathery type (Fig. 11A, 11B); 56–72 (holotype 67) in number, arranged in following formula (Selys-Longchamps 1907):

Composite formula


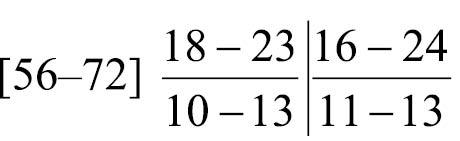


Mean formula


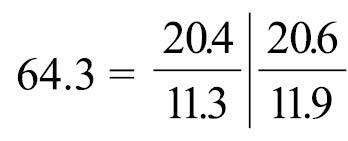


(n = 74 sections from 7 individuals)

Left and right lateral mesenteries present (Fig. 11A). Single giant nerve fiber, 15.98–36.03 μm in diameter (holotype avg. 27.40±6.29 μm, based on 5 sections from different parts of the body; avg. 25.93±6.05, based on 11 sections from different parts of the body, from five individuals [5 sections from holotype and 6 sections from 4 paratypes]), situated at base of left lateral mesentery (Fig. 11A, 11B). Esophageal valve absent.

Gonads not observed in any of our specimens; sex could thus not be determined.

#### Distribution and habitat.

*Phoronis emigi* is known only from a sandy bottom in northern Tomioka Bay, Amakusa, Japan, where we detected densities of up to about 90 individuals per 100 cm^2^. We observed no chitinous tubes after agitation and decantation during sampling, but the tubes would be fragile and might have been lost.

#### Remarks.

*Phoronis emigi* sp. n. is morphologically most similar to *Phoronis psammophila* Cori, 1889, with which it has in common 1) a long ascending branch of nephridium that is more than three times the length of the descending branch, 2) a single nephridial funnel, with the aperture situated at the tip of the descending branch, 3) a single giant nerve fiber situated on the left side, and 4) two lateral mesenteries. *Phoronis emigi* differs from *Phoronis psammophila* in the number of longitudinal muscle bundles in the body wall (56–72 vs. 25–50 in *Phoronis psammophila*) and the position of the right nephridiopores (at the same level as the anus vs. lower than the anus in *Phoronis psammophila*) (cf. Andrews 1890, Selys-Longchamps 1907, Marsden 1959, Long 1960, Emig 1968, 1971b, 1979).

Naturally, *Phoronis emigi* is morphologically similar to, but distinct from, the nominal *Phoronis architecta* Andrews, 1890, which is regarded as a junior synonym of *Phoronis psammophila* (Emig 1971b, 1974). Based on the descriptions by Andrews (1890) and Brooks and Cowles (1905), Emig (1971b, 1974) noticed that *Phoronis psammophila* and *Phoronis architecta* are morphogically identical, with the exception of the differences in larval brooding type and the presence of nidamental gland. Subsequently, Emig (1977) found that *Phoronis psammophila* shows a sympatric occurrence with *Phoronis muelleri* in the type locality of *Phoronis architecta*; therefore, he concluded that the larval brooding type and the absence of nidamental gland of *Phoronis architecta* described in Brooks and Cowles (1905) came from a specimen of *Phoronis muelleri*. On the other hand, some researchers have suggested the need of reexamination of the synonymy (Stancyk et al. 1976, Santagata and Zimmer 2002). Although we could not observe the larval brooding type of *Phoronis emigi*, the present species is clearly different from any of these species, *Phoronis psammophila*, *Phoronis muelleri*, and nominal *Phoronis architecta*, in the adult morphologies such as number of longitudinal muscle bundles.

The lack of gonads in our specimens was probably due to breeding seasonality. The breeding period of phoronid species previously studied is generally from spring to autumn (Rattenbury 1953, Emig 2003), whereas our material was collected at the end of November. Our specimens were likely in the post-breeding condition, following spawning and the relaease of embryos.

### Morphological analyses

**Figure 12.**
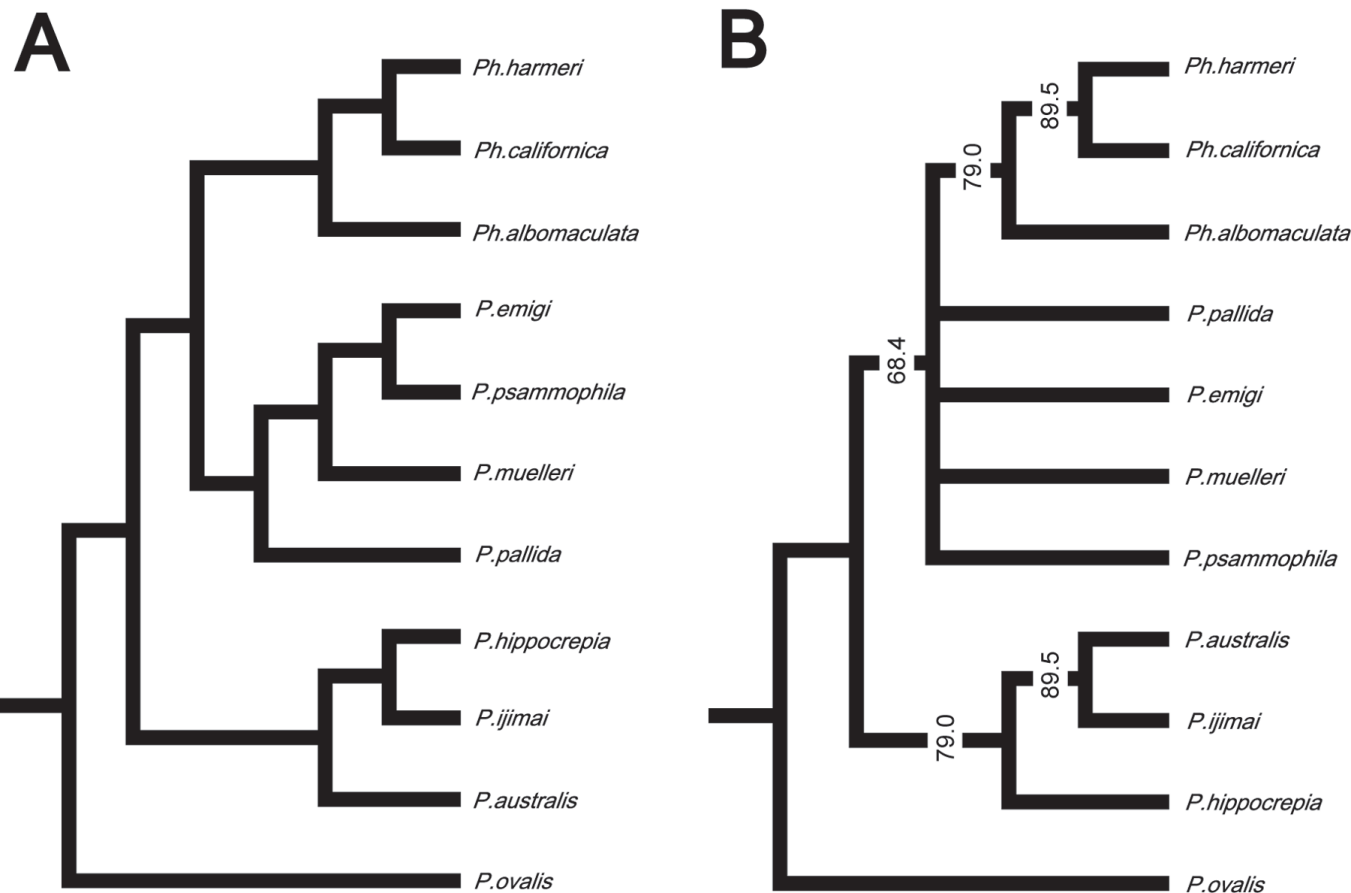
**A** Cladogram of single-linkage cluster analysis among 11 phoronid species based on 32 morphological characters **B** majority-rule consensus tree of 57 equally parsimonious tree obtained by cladistic analysis among 11 phoronid species based on 32 morphological characters. Numerals on nodes indicate frequency values.

In the resulting cladogram from the cluster analysis (Fig. 12A), three major clades were retrieved: 1) *Phoronopsis harmeri* + *Phoronopsis californica* + *Phoronopsis albomaculata*; 2) *Phoronis emigi* + *Phoronis psammophila* + *Phoronis muelleri* + *Phoronis pallida*; and 3) *Phoronis hippocrepia* + *Phoronis ijimai* + *Phoronis australis*. It shows the morphological similarity of the new species *Phoronis emigi* with *Phoronis psammophila*, sharing 16 adult morphological characters. *Phoronis emigi* also resembles *Phoronis muelleri* and *Phoronis pallida*, with which it shares 15 and 12 characters, respectively (Fig. 12A; Suppl. material 1). We conducted another cluster analysis without nephridial characters (eliminating character 6–14 in Suppl. material 1) to test the influence of the large amount of nephridial characters. In the resulting cladogram (Appendix 1 - Supplementary Fig. S1A), the same three major clades mentioned above were also obtained, although the topology between/within the three clades changed.

Our cladistic analysis yielded 57 equally parsimonious trees. The majority-rule consensus tree of those (Fig. 12B) did not resolve the relationship between *Phoronis emigi*, *Phoronis psammophila*, *Phoronis muelleri*, and *Phoronis pallida*; these four species formed a large clade together with *Phoronopsis* spp., with low consensus frequency value (68.4%). Another clade including three species (*Phoronis australis* + *Phoronis ijimai* + *Phoronis hippocrepia*) appeared as a sister group to this large clade; *Phoronis australis* formed a clade with *Phoronis ijimai* (89.5% in consensus frequency), to which *Phoronis hippocrepia* was the sister taxon (79.0% in consensus frequency). A parsimony tree without nephridial characters (Appendix 1 - Supplementary Fig. S1B) was almost identical to the tree including nephridial characters, except that *Phoronis emigi* appeared as sister to *Phoronopsis* (85.0% in consensus frequency), and *Phoronis ijimai* formed a clade with *Phoronis hippocrepia* (67.0% in consensus frequency).

### Molecular phylogeny

In this study, most of the sites for both 18S and 28S were unambiguously aligned; therefore, we used the entire region excluding gap sites for our phylogenetic analyses. For the COI dataset, we used all the codon positions in our phylogenetic analyses.

**Figure 13.**
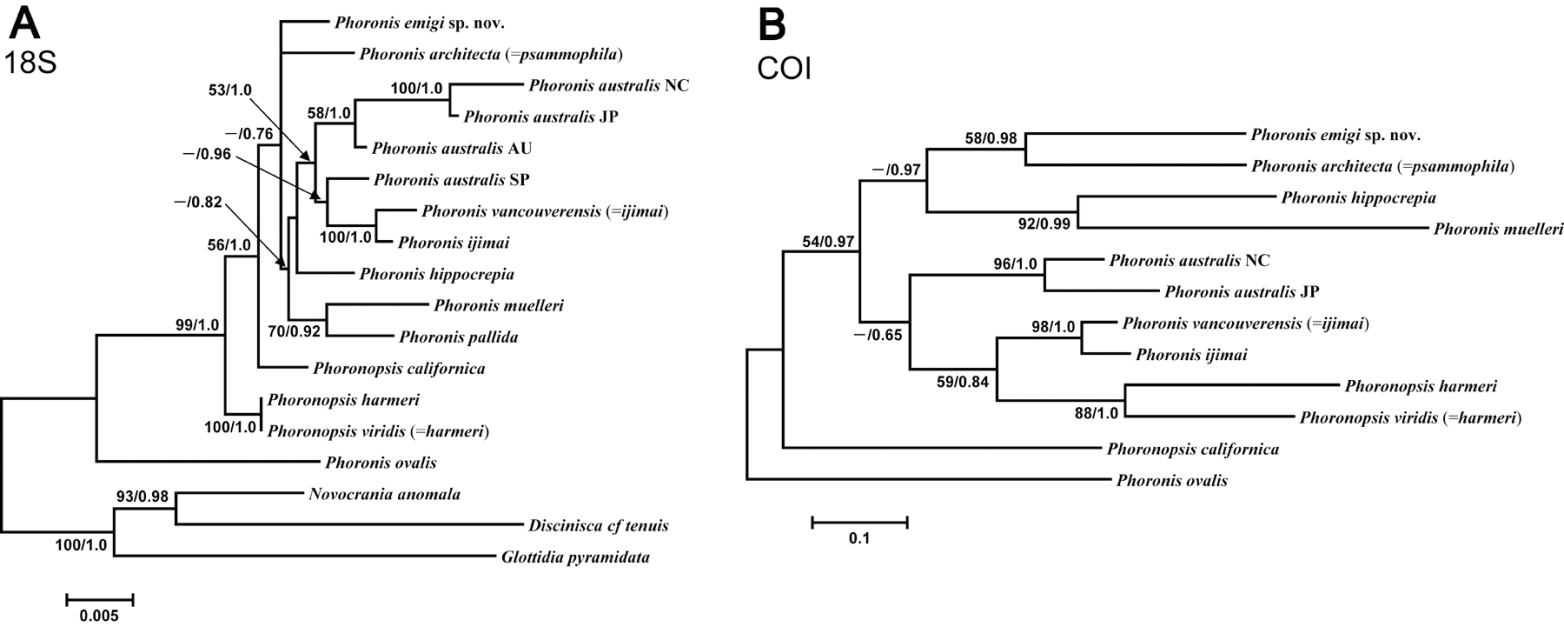
**A** Maximum-likelihood tree for 15 phoronid samples based on 18S data; three brachiopod species (*Novocrania anomala*, *Discinisca* cf. *tenuis*, and *Glottidia pyramidata*) are included as outgroup taxa **B** maximum-likelihood tree for 12 phoronid samples based on COI data; the tree is rooted with *Phoronis ovalis*. The scale bars indicate branch length in substitutions per site. Nodal support values are presented as the ML bootstrap value followed by the Bayesian posterior probability; only values >50% and 0.50, respectively, are shown.

The 18S dataset comprised 1756 bp aligned sites, with 208 variable sites, for 15 ingroup taxa. In the resulting ML tree (Fig. 13A) (log *L* = −4104.32), not all nodes are resolved or well supported. *Phoronis emigi* appears in a polytomous clade along with *Phoronis architecta* (= *psammophila*) and a large, weakly supported clade that includes *Phoronis ijimai* and nominal “*Phoronis vancouverensis*” from California. Japanese *Phoronis ijimai* is the sister taxon to nominal “*Phoronis vancouverensis*” from California, with high nodal support (100/1.0). These species are embedded in a clade otherwise containing only *Phoronis australis* from various localities, with Spanish *Phoronis australis* the sister taxon to the *ijimai*/“*vancouverensis*” clade (nodal support, - /0.96). The Bayesian tree (log *L* = −4371.60) was identical in topology to the ML tree.

The 28S dataset comprised 1065 bp aligned sites, with 333 variable sites, for 13 ingroup taxa. Most nodes in the ML tree (Appendix 1 - Supplementary Fig. S2) (log *L* = −3898.29) are resolved, and many have high nodal support. *Phoronis emigi* forms a clade with *Phoronis australis* from New Caledonia with moderate to high nodal support (97/0.71). *Phoronis australis* appears as polyphyletic, with nominal “*Phoronis vancouverensis*” comprising the sister taxon to a well-supported but polytomous clade containing *Phoronis australis* from Australia and Japan, and *Phoronis muelleri*. We did not obtain a 28S sequence for *Phoronis ijimai*, which is thus missing from this analysis. The resulting Bayesian tree (log *L* = −4601.76) is topologically identical with the ML tree, but the clade containing *Phoronis emigi* and New Caledonian *Phoronis australis* is supported by lower Bayesian posterior probability (0.71).

The COI dataset comprised 621 bp aligned sites, with 253 variable sites, for 12 ingroup taxa (the tree was rooted with *Phoronis ovalis*, which was the basal phoronid in all trees rooted with brachiopods). The resulting ML tree (Fig. 13B) (log *L* = −3633.85) is completely resolved, but with variable nodal support. The sister taxon to *Phoronis emigi* is *Phoronis architecta* (= *psammophila*) rather than New Caledonian *Phoronis australis* as in the 28S ML tree. The two *Phoronis australis* samples inlcuded in the analysis form a clade with high support (96/1). *Phoronis ijimai* and nominal “*Phoronis vancouverensis*” group together with high support (98/1), with this clade forming the sister group (nodal support, 59/0.84) to (*Phoronopsis harmeri* + *Phoronopsis viridis*). *Phoronopsis* appeared polyphyletic, with *Phoronopsis californica* the sister taxon to all other phoronids except *Phoronis ovalis*. The resulting Bayesian tree (log *L* = −3772.71) was identical in topology to the ML tree.

**Figure 14.**
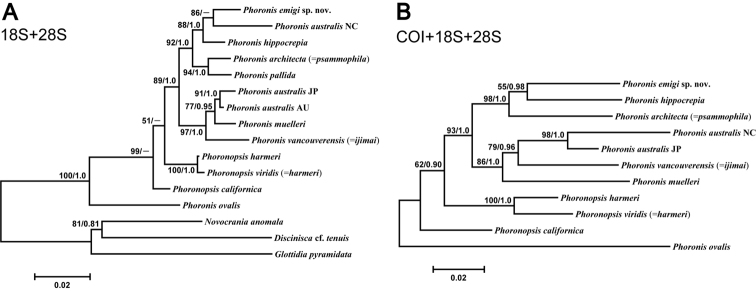
**A** Maximum-likelihood tree for 13 phoronid samples based on the combined 18S + 28S data set; three brachiopod species (*Novocrania anomala*, *Discinisca* cf. *tenuis*, and *Glottidia pyramidata*) are included as outgroup taxa **B** maximum-likelihood tree for 11 phoronid samples based on the combined COI + 18S + 28S data set; the tree is rooted with *Phoronis ovalis*. Scale bars indicate branch length in substitutions per site. Nodal support values are presented as the ML bootstrap value followed by the Bayesian posterior probability; only values >50% and 0.50, respectively, are shown.

The concatenated 18S–28S dataset comprised 2819 bp aligned sites, with 537 variable sites, for 13 ingroup taxa. The ML tree (Fig. 14A) (log *L* = −8247.64) was identical in topology to the 28S ML tree (Appendix 1 - Supplementary Fig. S2), except the unresolved trichotomy of AU and JP *Phoronis australis* and *Phoronis muelleri* in the latter is resolved in the 18S-28S tree. The Bayesian tree (log *L* = −9181.86) differs from the ML tree in that *Phoronis emigi* forms a clade with *Phoronis hippocrepia*, with New Caledonian *Phoronis australis* the sister group to this clade.

The concatenated 18S–28S–COI dataset comprised 3440 bp aligned sites, with 555 variable sites, for 11 ingroup taxa (the tree was rooted with *Phoronis ovalis*). The resulting ML tree (Fig. 14B) (log *L* = −10594.85) differs from the 28S and 18S–28S trees in several ways. The sister taxon to *Phoronis emigi* is *Phoronis hippocrepia* (nodal support, 55/0.98) rather than New Caledonian *Phoronis australis*. The positions of New Caledonian *Phoronis australis* and *Phoronis architecta* (= *psammophila*) are different in the 18S–28S–COI ML tree, but these changes in topology appear to some extent due to the omission of *Phoronis pallida* from the 18S–28S–COI dataset. The topology within the “*Phoronis vancouverensis*” / *Phoronis australis* / *Phoronis muelleri* clade also differs between 18S–28S–COI ML and the other trees that include 28S. The 18S–28S–COI Bayesian tree (log *L* = −10802.56), was identical to the ML tree in topology.

## Discussion

Before our study, three species of phoronids had been recorded from Japan: *Phoronis ijimai*, *Phoronis australis*, and *Phoronis psammophila*. The former two were reported from Misaki (Oka 1897, Ikeda 1902), and the latter from Lake Hamana (Hirose et al. 2011). *Phoronis ijimai* was also reported from Akkeshi under the name *Phoronis hippocrepia* (Uchida and Iwata 1955), but the taxonomic identity of this population is uncertain (Hirose et al. 2011). Bailey-Brock and Emig (2000) listed Tokyo Bay as a locality for *Phoronis pallida*, with the note “coll. T. Furota”, although they did not include any other details about the specimens. The known phoronid diversity in Japan thus remains low, with all specimens reported from sandy substratum. Investigations on rocky shores may yield additional species in the future.

**Figure 15.**
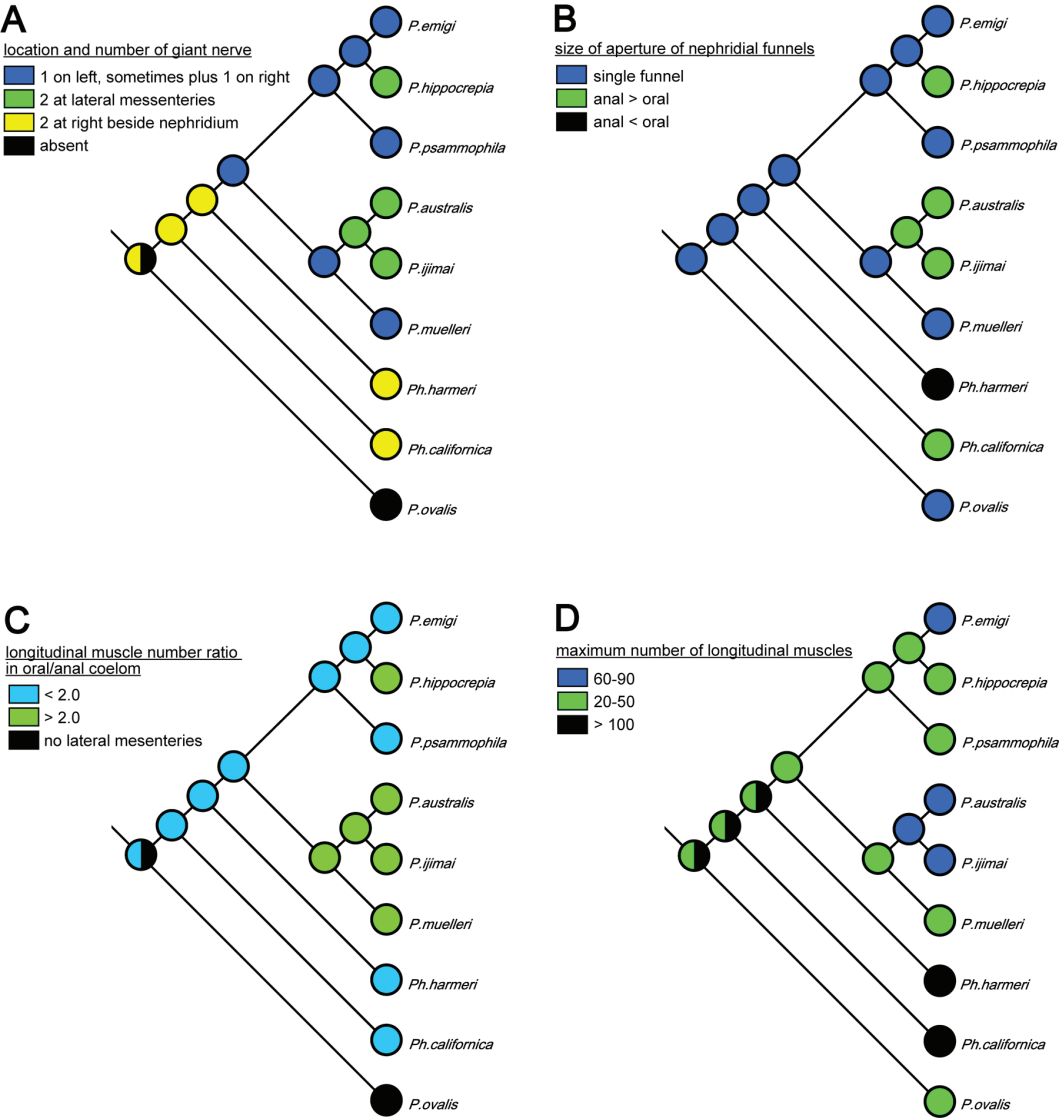
Parsimonious reconstruction of four adult morphological characters among nine phoronid species on the maximum-likelihood tree based on concatenated COI–18S–28S dataset.

Although the molecular phylogenetic trees (Figs 13A, 13B, 14A, 14B; Appendix 1 - Supplementary Fig. S2) produced by the various datasets differed in topology, our phylogenetic reconstructions suggest that most of the adult morphological characters used to date in phoronid taxonomy are highly homoplastic (Fig. 15A–D), and thus phylogenetically less informative than the molecular data. According to the character matrix and the cladogram based on 32 morphological and reproductive characters among 11 phoronid species (Suppl. material 1; Fig. 12A, 12B; Appendix 1 - Supplementary Figs S1A, S1B, S3A–D, S4A–D), *Phoronis emigi* comprise a group with *Phoronis psammophila*, *Phoronis muelleri*, and *Phoronis pallida*. In none of our molecular trees (Figs 13A, 13B, 14A, 14B), however, did these four species alone comprise a clade. In the COI tree (Fig. 13B), *Phoronis architecta* (= *psammophila*), *Phoronis muelleri*, and *Phoronis emigi* comprise a clade that also includes *Phoronis hippocrepia*. In the COI–18S–28S tree (Fig. 14B), *Phoronis emigi* and *Phoronis architecta* (= *psammophila*) group with *Phoronis hippocrepia*, to the exclusion of *Phoronis muelleri*, but no morphological or reproductive characters (Suppl. material 1; Fig. 15) appear to be synapomorphic for this clade, though character 19 (ratio of number of longitudinal muscles in oral coelom / anal coelom) in these three species is smaller than in other species of the genus except for *Phoronis ovalis*, which lacks lateral mesenteries (Suppl. material 1).

Our molecular trees do not correspond with any of the subdivisions of phoronids suggested by previous researchers solely based on morphological characters (Silén 1952, Marsden 1959, Emig 1974). Within the phylum, Emig (1974) proposed five subgroups based on nephridial structure (Appendix 1 - Supplementary Fig. S5); most of these subgroups were identical to those in Silén’s (1952) morphological categorization, except that Silén (1952) grouped *Phoronis psammophila* with *Phoronis ijimai* rather than *Phoronis muelleri*. Although relationships within each group vary depending on the characters used in the analyses, our morphology-based cladograms (Fig. 12; Appendix 1 - Supplementary Figs S1, S3, S4) mostly correspond Emig’s (1974) subgroup relationships; therefore, Emig (1974) would have been classified *Phoronis emigi* in his “group 3” along with *Phoronis psammophila* and *Phoronis muelleri* based on nephridial morphology. None of our molecular trees (Figs 13A, 13B, 14A, 14B; Appendix 1 - Supplementary Fig. S2), however, shows a clade comprising these three species alone. In the COI tree (Fig. 13B), these species form a clade that also includes *Phoronis hippocrepia*.

Our morphological and molecular results do not contradict that “*Phoronis vancouverensis*” is conspecific with *Phoronis ijimai*, as proposed by Emig (1971a). Although we were not able to obtain a 28S sequence for *Phoronis ijimai*, in the 18S and COI trees it always formed a clade with “*Phoronis vancouverensis*” accompanied by high nodal support (Fig. 13A, 13B). The Kimura (1980) 2-parameter (K2P) distance between *Phoronis ijimai* and “*Phoronis vancouverensis*” for 583 bp of COI was 0.07, substantially below the value of the intraspecific distance 0.115 between *Phoronis australis* NC and *Phoronis australis* JAPAN (Table 2). On the other hand, the interspecific distances among phoronids ranged from 0.164 to 0.287; therefore, K2P divergence factor between 0.115 and 0.164 could be a threshold for discriminating phoronid species.

### Taxonomic key to Japanese Phoronida

**Table:** 

1	Inhabiting cerianthid tube-wall; lophophore multispiral; normally black in color	*Phoronis australis* Haswell, 1883
–	Inhabiting cylindrical tube on hard substrate or soft sandy and muddy bottom; lophophore horseshoe-shaped without significant coiling; white or red in color	2
2	Cylindrical tube constructed of small sand grains; tentacles fewer than 100 in number, with white spots	*Phoronis psammophila* Cori, 1889
–	Cylindrical tube obscure or not constructed of sand grains; tentacles more than 100 in number, without white spots	3
3	Left giant nerve fiber more than 15 µm in diameter, right giant nerve fiber absent; longitudinal muscles of feathery type, more than 10 in number on each side of anal coelom; nephridium with single funnel, nephridial papilla absent, descending branch present	*Phoronis emigi* sp. n.
–	Left giant nerve fiber less than 15 µm in diameter, right giant nerve fiber present; longitudinal muscles of bushy type, fewer than 10 in number on each side of anal coelom; nephridium with two funnels, nephridial papilla present, descending branch absent	*Phoronis ijimai* Oka, 1897

**Table 2.** Pairwise genetic distances (K2P distances) based on 583 positions of COI sequences between *Phoronis ijimai*, *Phoronis emigi*, and the other species. The largest (*Phoronis australis* JP and *Phoronis muelleri*) and the lowest (*Phoronis australis* NC and *Phoronis vancouverensis*) interspecific distances are also listed. The analysis involved 12 phoronid sequences.

Species 1	Species 2	K2P Distance
*Phoronis australis* JP	*Phoronis muelleri*	0.287
*Phoronis australis* NC	*Phoronis vancouverensis*	0.164
*Phoronis australis* NC	*Phoronis australis* JAPAN	0.115
*Phoronis ijimai*	*Phoronis muelleri*	0.278
	*Phoronis architecta*	0.258
	*Phoronopsis californica*	0.258
	*Phoronis ovalis*	0.239
	*Phoronis hippocrepia*	0.222
	*Phoronopsis viridis*	0.216
	*Phoronopsis harmeri*	0.215
	*Phoronis australis* JAPAN	0.206
	*Phoronis australis* NC	0.179
	*Phoronis vancouverensis*	0.070
*Phoronis emigi* sp. n.	*Phoronis muelleri*	0.274
	*Phoronopsis viridis*	0.259
	*Phoronopsis harmeri*	0.252
	*Phoronis ovalis*	0.240
	*Phoronis hippocrepia*	0.239
	*Phoronopsis californica*	0.238
	*Phoronis ijimai*	0.235
	*Phoronis vancouverensis*	0.218
	*Phoronis australis* JAPAN	0.208
	*Phoronis australis* NC	0.205
	*Phoronis architecta*	0.202

## Supplementary Material

XML Treatment for Phoronis ijimai

XML Treatment for Phoronis emigi

## Figures and Tables

**Supplementary Figure S1.**
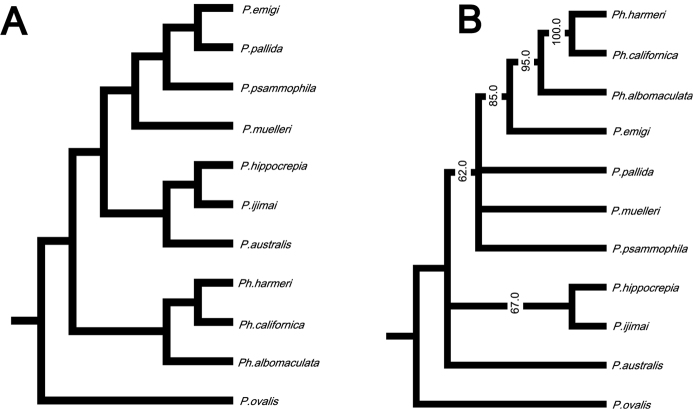
**A** Cladogram of single-linkage cluster analysis among 11 phoronid species based on 23 morphological characters excluding nephridial characters **B** majority-rule consensus tree of 100 equally parsimonious tree obtained by cladistics analysis among 11 phoronid species based on 23 morphological characters excluding nephridial characters. Numerals on nodes indicate frequency values.

**Supplementary Figure S2.**
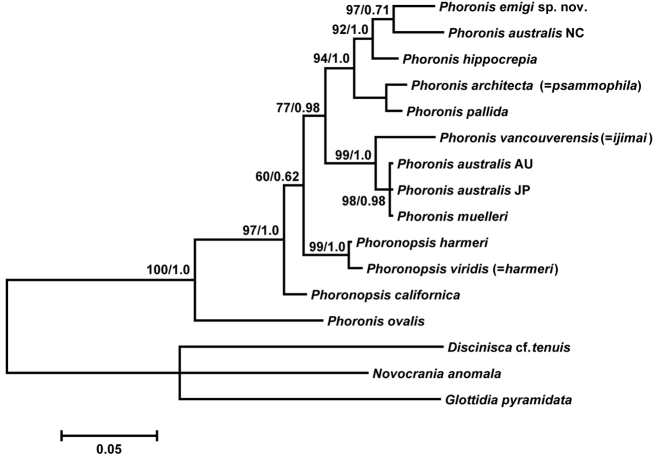
Maximum-likelihood tree for 13 phoronid samples based on 28S data; three brachiopod species (*Novocrania anomala*, *Discinisca* cf. *tenuis*, and *Glottidia pyramidata*) are included as outgroup taxa. The scale bar indicates branch length in substitutions per site. Nodal support values are presented as the ML bootstrap value followed by the Bayesian posterior probability; only values >50% and 0.50, respectively, are shown.

**Supplementary Figure S3.**
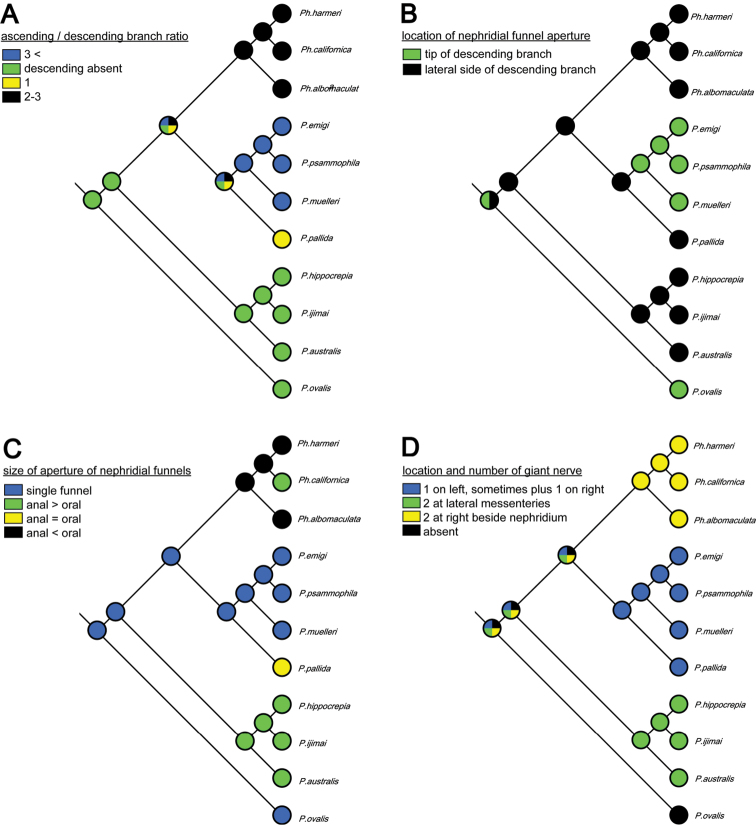
Parsimonious reconstruction of four adult morphological characters among 11 phoronid species on the cladogram of the cluster analyses based on 32 morphological characters.

**Supplementary Figure S4.**
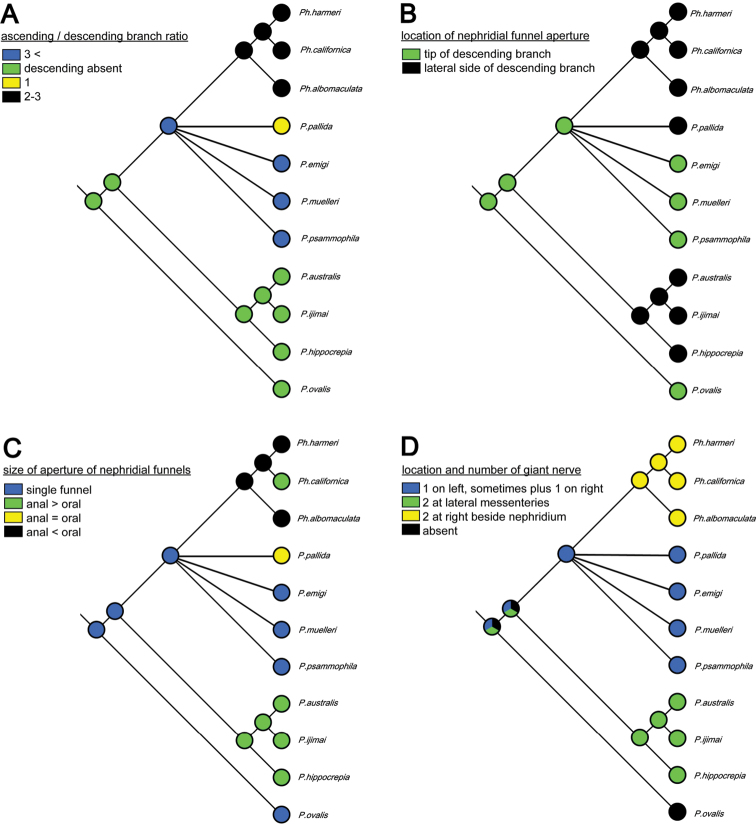
Parsimonious reconstruction of four adult morphological characters among 11 phoronid species on the parsimonious consensus tree based on 32 morphological characters.

**Supplementary Figure S5.**
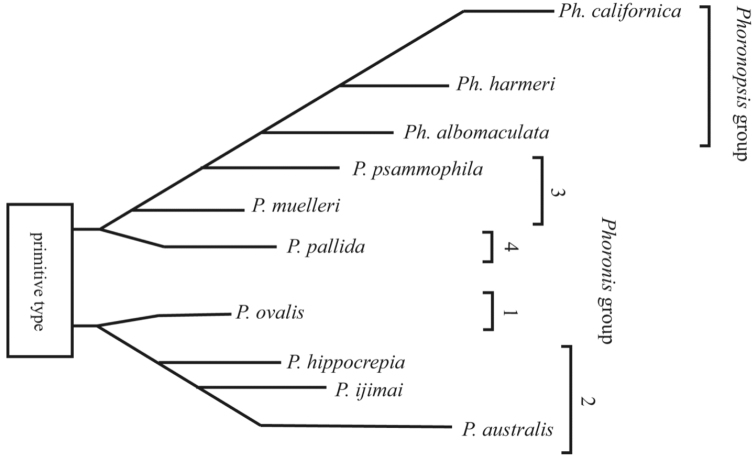
Emig’s (1974) classification of five morphological categories within Phoronida, based on nephridial structure. Modified from Emig (1974).

## Supplementary material

Supplementary material 1 Character matrix of 32 morphological and reproductive characters among 11 phoronid species considered in the Discussion. doi: 10.3897/zookeys.398.5176.app1 Data type: character matrix This dataset is made available under the Open Database License (http://opendatacommons.org/licenses/odbl/1.0/). The Open Database License (ODbL) is a license agreement intended to allow users to freely share, modify, and use this Dataset while maintaining this same freedom for others, provided that the original source and author(s) are credited.Masato Hirose, Ryuma Fukiage, Toru Katoh, Hiroshi Kajihara
